# Complexes Formed by the K63-Specific Deubiquitinating Enzyme BRCC36: New Promising Therapeutic Targets in Human Disease

**DOI:** 10.3390/biom15121724

**Published:** 2025-12-11

**Authors:** Xinyu Zhang, Xiaodong Pang, Yili Chen, Yue Liu, Jian-An Huang, Yuanyuan Zeng

**Affiliations:** 1 Department of Pulmonary and Critical Care Medicine, The First Affiliated Hospital of Soochow University, Suzhou 215006, China; 20244232020@stu.suda.edu.cn (X.Z.); 20245232033@stu.suda.edu.cn (X.P.); 20234232021@stu.suda.edu.cn (Y.C.); 20254232019@stu.suda.edu.cn (Y.L.); jahuang@suda.edu.cn (J.-A.H.); 2 Institute of Respiratory Diseases, Soochow University, Suzhou 215006, China

**Keywords:** Deubiquitination, BRCC36, BRISC, BRCA1, SHMT2

## Abstract

BRCC36, a member of the JAB1/MPN/Mov34 metalloenzymes family, exhibits distinct biochemical characteristics compared to other monomeric deubiquitinating enzymes. To function as a deubiquitinating enzyme, BRCC36 must assemble into a complex with other subunits that specifically cleaves K63-linked polyubiquitin chains. In the cytoplasm, BRCC36 forms the BRISC complex, which plays a crucial role in regulating various signaling pathways through modulating the K63-linked ubiquitination of substrate proteins. The BRISC complex can interact with the cytoplasmic SHMT2, thereby influencing diverse biological processes, including inflammation, mitosis, and hematopoiesis. Within the nucleolus, BRCC36 forms the BRCA1-A complex, which contributes to DNA damage repair. Growing evidence underscores the importance of the ubiquitin system, particularly deubiquitinating enzymes, in the initiation and progression of various diseases. In this review, we first provide a comprehensive overview of the localization, assembly, mutations, and functions of BRCC36 and its associated complexes. We then discuss recent advances in research on BRCC36 across various diseases and explore its potential as a therapeutic target.

## 1. Introduction

Ubiquitination is a pivotal post-translational modification that regulates a wide range of biological processes. This modification can be reversed by the deubiquitinating enzymes (DUBs), which catalyze the removal of ubiquitin from substrate proteins or polyubiquitin chains [1,2]. The human gene BRCC3 encodes the lysine-63-specific deubiquitinase BRCC36, which is ubiquitously expressed across various tissues and involved in diverse cellular activities [3]. The majority of DUB families belong to the cysteine protease, with the notable exception of the JAB1/MPN/Mov34 metalloenzymes (JAMM) family, whose members function as zinc-dependent metalloproteases [4,5]. BRCC36 can assemble into complexes with other subunits, such as the BRISC or BRCA1-A complexes, to execute its deubiquitinating activity [5,6,7,8,9].

BRCA1-A and BRISC are two distinct multi-protein complexes that share several core components, including MERIT40, BRCC36, and BRE. A key compositional distinction between them lies in the scaffold protein associated with BRCC36. Within the BRCA1-A complex, BRCC36 interacts with ABRAXAS, whereas in the BRISC complex, it is scaffolded by ABRO1 (Table 1) [7,10,11]. Although both complexes exhibit DUB activity and possess common subunits, they fulfill divergent biological roles and operate through distinct molecular mechanisms [11]. For instance, BRCA1-A is primarily implicated in DNA damage response and repair, whereas BRISC plays a critical role in the regulation of immune signaling processes [6,12,13].

This review systematically summarizes recent advances in understanding the roles of BRCC36 in various pathological conditions and explores its underlying molecular mechanisms. We begin by introducing the general concept of ubiquitination, with a particular focus on the pathological significance of DUBs in disease development and progression. Next, we detail the structural characteristics, subcellular localization, and functional implications of specific mutations of BRCC36. The review further describes the molecular processes through which BRCC36 assembles into multi-subunit complexes, specifically the BRCA1-A and BRISC complexes. In light of the critical functional interplay between serine hydroxymethyltransferase 2 (SHMT2) and the BRISC complex, we also discuss the context-dependent associations of SHMT2, considering its diverse subcellular localizations and structural isoforms, within BRISC-mediated pathways. Finally, we analyze the emerging roles of BRCC36 in oncogenesis, cardiovascular diseases, immune-related disorders, and some other diseases. It is anticipated that this comprehensive overview will not only deepen the understanding of BRCC36 and its associated complexes but also facilitate future investigations into structure-to-function relationships of key molecular domains, complex assembly mechanisms, and the development of targeted therapeutic strategies along with clinically applicable diagnostic biomarkers.

## 2. The Research Progress of Ubiquitination in Diseases

**Figure 1:**
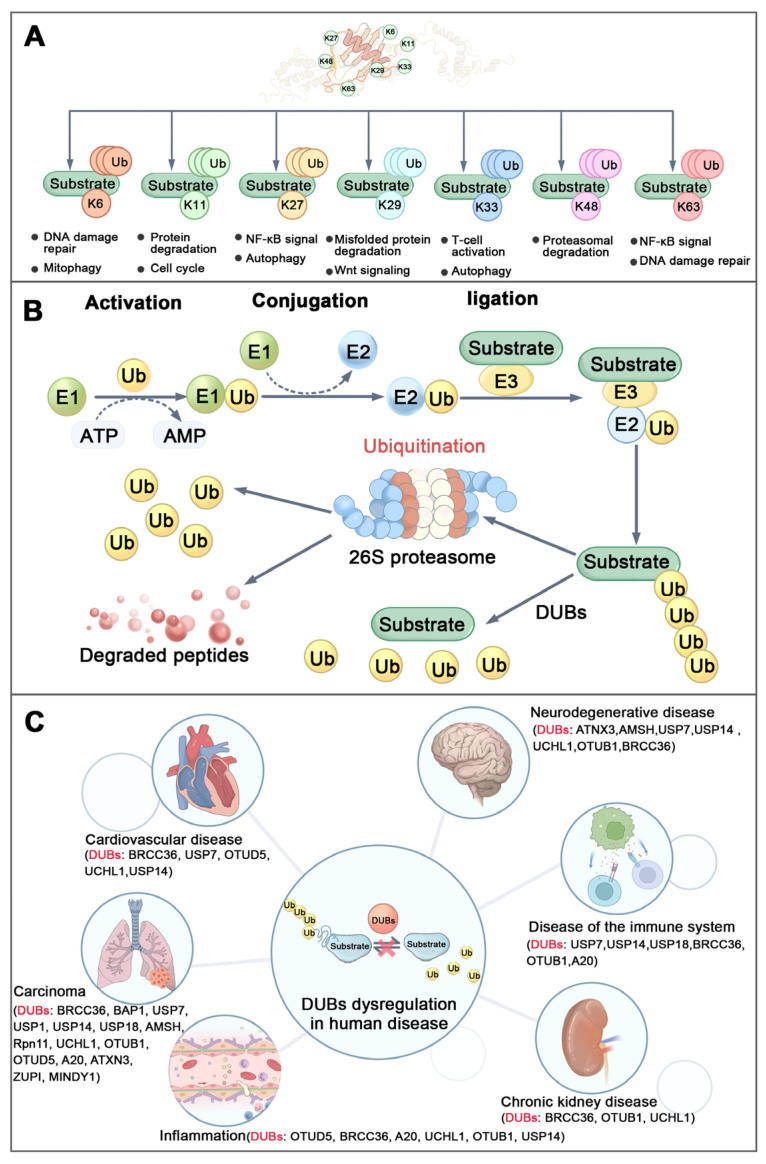
Ubiquitination and diseases. (**A**) Structure and function of different types of ubiquitin chains. (**B**) Brief process of ubiquitination: Ubiquitin-activating enzyme (E1) activates the ubiquitin molecule in ATP-dependent way. Then the activated ubiquitin is transferred from E1 to ubiquitin-conjugating enzyme (E2) to form the Ub-E2 complex. Finallly, ubiquitin ligase (E3) recognizes the target protein and transfers the ubiquitin from E2 to lysine residues of the target protein to complete the ubiquitination modification. (**C**) DUB dysregulation in human diseases. (Ub-Ubiquitin, DUBs-Deubiquitinating Enzymes).

The covalent modification of substrate proteins through the attachment of ubiquitin to lysine (K) residues represents a fundamental and widespread regulatory mechanism in eukaryotic cells [23,24]. Ubiquitin contains seven lysine residues (K6, K11, K27, K29, K33, K48, and K63), which serve as potential sites for chain formation [24,25]. A defining characteristic of ubiquitination is the capacity of ubiquitin molecules to polymerize through linkages via lysine residues or the N-terminal methionine, resulting in structurally and functionally distinct polyubiquitin chains (Figure 1A) [24,26,27]. Among these, the K48-linked polyubiquitin chain is the most prevalent, which is recognized as the signal for targeting proteins to the proteasome for degradation. In contrast, the K63-linked polyubiquitin chain plays crucial roles in non-proteolytic activities, including the regulation of cellular signaling pathways, DNA damage response, endosomal trafficking, and autophagy [23,28,29,30]. Notably, BRCC36 selectively targets and cleaves K63-linked polyubiquitin chains to regulate their functions [8].

The ubiquitin-mediated protein degradation pathway is catalyzed by a three-enzyme cascade comprising E1 ubiquitin-activating enzymes, E2 ubiquitin-conjugating enzymes, and E3 ubiquitin ligases (Figure 1B) [29,31]. This process is dynamically counterbalanced by DUBs, a specialized group of proteases that reverse ubiquitination. DUBs either cleave the peptide bond between the C-terminus of ubiquitin and N-terminus of the methionine of ubiquitin (in linear ubiquitin chains) or the isopeptide bond between the C-terminus of ubiquitin and the epsilon-amino group (side chain) of the lysine residue of the substrate protein or lysine-linked ubiquitin chain [4,32]. The human genome encodes more than 100 DUBs, which are classified into seven distinct families based on their sequence and structural homology (Table 2) [33,34]. Dysregulation of DUBs activity or expression can disrupt ubiquitin homeostasis, leading to loss of proteostatic balance and contributing to the pathogenesis of various diseases (Figure 1C) [31,35,36,37]. It is noteworthy that some DUBs exhibit high substrate specificity and depend on interaction with binding partners into complexes for full regulatory activity and biological function [7,38,39].

## 3. The Structure and Function of BRCC36

### 3.1. Structure of BRCC36

BRCC36 is a zinc-dependent metalloprotease [8]. The center of the active site is formed by a zinc ion, which is coordinated by two histidine residues (H122 and H124), one aspartate residue (D135), and a H_2_O molecule [8,9,10]. It is localized in both the nucleus and the cytoplasm and serves as the catalytic subunit responsible for the K63-linked deubiquitination in both compartments [6,11,48]. The enzymatic activity of BRCC36 is modulated by its associated scaffold proteins, which regulate not only its subcellular localization but also catalytic function [8,58]. Existing as the homodimer, isolated BRCC36 is inactive, while it displays DUB activity within a protein complex (such as the BRCC36-KIAA0157 heterodimer) [8]. This regulatory mode may be necessary due to the notable disparity between the number of E3 ubiquitin ligases (about 600) and DUBs (about 100) encoded in the human genome, suggesting that DUB activity is often controlled by partnerships with different regulatory proteins [29,59,60]. For example, in the cytoplasm, BRCC36 interacts with ABRO1 to form the BRISC complex [8,11]. ABRAXAS, a nuclear protein, which shares sequence homology with ABRO1, directs the nuclear localization of BRCC36 and facilitates its incorporation into the BRCA1-A complex [61,62]. This nuclear complex includes additional components such as RAP80, BRE, and MERIT40, and plays critical roles in DNA damage response and repair [63,64]. Notably, reduced expression of cytoplasmic ABRO1 leads to increased formation of the nuclear BRCA1-A complex, suggesting a dynamic balance between these two complexes in vivo [6].

### 3.2. Assembly of BRCA1-A Complex

**Figure 2:**
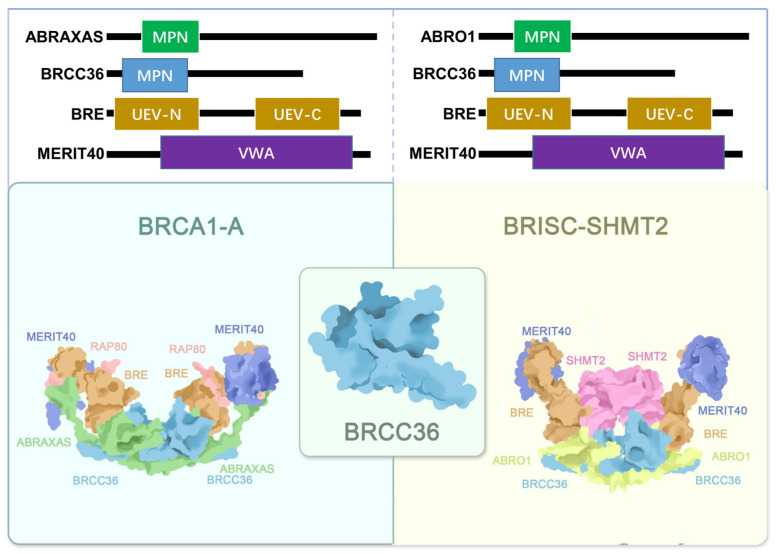
Structural sketch of the BRCA1-A (PDB ID: 6GVW) and BRISC-SHMT2 (PDB ID: 6H3C) complexes. The BRE, MERIT40, BRCC36, ABRAXAS, RAP80, ABRO1 and SHMT2 are colored orange, navy blue, light blue, green, pink, yellow and purple, separately.

The BRCA1-A complex exhibits a dimeric architecture of pentamers with overall C2 symmetry [9]. It is composed of two heteropentameric subunits, each comprising BRCC36, ABRAXAS, BRE, RAP80, and MERIT40, which assemble into an arched structure [8,10]. These two half-arcs contact each other primarily through BRCC36 and its scaffold protein ABRAXAS [19,65]. The core of the complex adopts a twisted V-shaped conformation formed by two ABRAXAS/BRCC36/BRE/MERIT40 tetramers [9,10]. Within each arm, the ABRAXAS/BRCC36 heterodimer localizes to the base of the V-shaped structure, while the BRE/MERIT40 pair extends along each arm [2,66] (Figure 2).

Although both BRCC36 and ABRAXAS contain an MPN domain, only the MPN domain of BRCC36 is catalytically active [10,67,68]. The MPN domain of ABRAXAS, however, plays an essential structural role by connecting the catalytic BRCC36 subunit to the distal end of the arc formed by BRE, MERIT40, and RAP80 [9,65,69]. ABRAXAS also serves as a central adaptor that links BRCA1 to the rest of the complex via phosphorylation-dependent interactions. Its C-terminal phosphorylated serine residue at position 406 (pSer406) mediates direct binding to the BRCT domains of BRCA1 [10,70].

BRE acts as a structural bridge between the ABRAXAS-BRCC36 MPN domain dimer and MERIT40, which contains a von Willebrand factor A (VWA) domain [7,19]. RAP80 is deeply integrated into the complex through extensive interactions with the other subunits [10,71]. It contains a dedicated domain that binds ABRAXAS and is critical for its association with ABRAXAS, BRCA1, and BRCC36 [64,72]. As a core structural component of BRCA1-A, RAP80 not only interacts with ABRAXAS but also exhibits substantial interaction interfaces with both MERIT40 and BRE [21,48,69].

Recent research by Tang (2024) revealed that DOT1L-mediated methylation of RAP80 plays a key role in recruiting the BRCA1-A complex to chromatin for DNA damage repair [64]. The same study also identified MERIT40 as an integral component of the BRCA1-A complex, essential for maintaining the stability of BRE and ABRAXAS, as well as for the recruitment of BRCA1 to DNA damage sites [19].

### 3.3. Assembly of BRISC Complex

The BRISC complex is a U-shaped dimer composed of four subunits and exhibits distinct C2 symmetry [66]. Its architecture shares notable similarities with the BRCA1-A complex [66]. The arrangement of the JAMM/MPN domains in ABRO1 and BRCC36 is conserved between the two complexes, as are the interfaces mediating dimerization [9,10]. However, significant conformational differences exist in the UEV-C domain of BRE and the orientation of MERIT40, which is rotated by approximately 56° compared to its position in the BRCA1-A crystal structure [9,10,66]. Notably, in BRISC, the absence of RAP80, the subunit that in BRCA1-A extensively interacts with ABRO1, MERIT40, and BRE, leaves the super-tetramer surface of BRE exposed, resulting in a more flexible overall structure [9,10,67,73].

SHMT2 is widely recognized as a metabolic enzyme, and most early studies focused on its canonical catalytic functions [74,75]. It exists in two major isoforms: mitochondrial SHMT2, which localizes to mitochondria, and cytosolic SHMT2α, which lacks the mitochondrial targeting sequence [66,76,77]. In recent years, non-catalytic roles of SHMT2 have gained increasing attention [78]. Particularly noteworthy is the BRISC-SHMT2 complex, in which SHMT2 serves as a fifth structural component (Figure 2) [13]. SHMT2 binds the complex as a dimer, positioned between the two arms of BRISC directly above the BRCC36-ABRO1 super-dimer. It interacts via its α6 helix with the MPN domains of BRCC36 and ABRO1, as well as with the UEV-N and UEV-M domains of BRE. This interaction mediates the structural integration of SHMT2 into the complex [9,10,66].

Structural and functional analyses indicate that SHMT2 binding sterically occludes the catalytic site of BRCC36, thereby inhibiting the deubiquitinating activity of the BRISC complex [9,10,66]. Structural studies further reveal that the dimeric form of SHMT2, specifically the cytosolic isoform, is exclusively incorporated into BRISC [66,78]. Lysine 280 (K280) in SHMT2 and intracellular concentrations of pyridoxal phosphate (PLP), which promotes tetramerization of SHMT2, critically regulate its oligomeric state. Mutation at K280 impedes tetramer formation, leading to accumulation of dimeric SHMT2 and enhanced incorporation into BRISC, thereby promoting deubiquitination. Conversely, PLP stimulation shifts SHMT2 toward the tetrameric state, favoring its metabolic functions over its role in BRISC-mediated signaling [66,76,79,80]. The precise mechanism by which dimeric SHMT2 participates in BRISC-mediated substrate deubiquitination remains unknown. It has been proposed that the proximity of a K63-linked polyubiquitin substrate may competitively displace SHMT2 from BRISC, thereby promoting deubiquitination, suggesting a potential auto-regulatory mechanism for BRCC36 DUB activity [66,81].

### 3.4. Function of BRCC36

BRCC36 is a metalloprotease that specifically cleaves K63-linked polyubiquitin chains [5,6]. Rather than functioning as a simple DUB, it acts as a multifunctional regulatory DUB whose activity is tightly controlled by spatially specific incorporation into either the BRCA1-A or BRISC complexes and selective engagement with K63-linked ubiquitin substrates [5,6,8]. Through these mechanisms, BRCC36 plays central roles in critical biological processes, including DNA repair, immune regulation, and cell death [13,66,82].

As part of the BRCA1-A complex, BRCC36 specifically recognizes histone H2A and H2AX conjugated with K63-linked ubiquitin chains at DNA damage sites. This activity facilitates the recruitment of the BRCA1-BARD1 (BRCA1-associated RING domain protein 1) heterodimer to double-strand breaks (DSBs) and antagonizes RNF8-dependent ubiquitination at these loci [19,82,83,84]. Notably, BRCA1 partners with BARD1 and additional tumor-suppressor proteins to exert critical functions in DNA repair, replication-fork maintenance, and tumor suppression [85]. Within the BRISC complex, BRCC36 is essential for proper mitotic spindle assembly and kinetochore-microtubule attachment [86]. BRCC36 also regulates interferon (IFN) signaling by deubiquitinating the IFN receptor, IFNAR1, thereby enhancing its stability and cell surface expression [8,13]. Furthermore, it modulates inflammatory responses by mediating the deubiquitination of NLRP3 (Nucleotide-binding oligomerization domain-like receptor family pyrin domain-containing 3), which promotes NLRP3 inflammasome assembly, and downregulates cellular responses to bacterial lipopolysaccharide (LPS) via its action on IFNAR1 [87]. These diverse functions underscore the central regulatory role of BRCC36 within different macromolecular complexes [66].

Recent advances have enabled functional mapping of key residues in BRCC36. The high-order interaction between BRCC36 and ABRO1 is required for its DUB activity and for interactions with partner proteins such as SHMT2 and RAP80 [8,9]. Mutations at residues L23 and/or L27 disrupt BRCC36’s binding to ABRO1, impair DUB activity, compromise its localization to DNA damage sites, and abrogate interactions with ABRO1, RAP80, SHMT2, BRE, and MERIT40 [8]. Additionally, mutations at F52 or the metal-coordinating residues H122/H124 abolish deubiquitination activity, with H122/H124 mutations also eliminating metalloprotease activity and DNA repair function [5,83,84,86,88]. The A278 mutation disrupts interactions with RAP80 and SHMT2. As research progresses, the mutational landscape of BRCC36 is expected to provide new targets and biomarkers for personalized therapies in related diseases [8].

## 4. Advances in the Research of BRCC36 in Human Diseases

### 4.1. Roles of BRCC36 in Carcinoma

#### 4.1.1. Hepatocellular Carcinoma

Hepatocellular carcinoma (HCC) is one of the most prevalent causes of cancer-related deaths worldwide and is characterized by high heterogeneity, poor prognosis, and limited response to pharmacological interventions [89,90]. Tao’s research identified BRCC36 as a DUB for 3-hydroxy-3-methylglutaryl-CoA reductase (HMGCR), which is the rate-limiting enzyme of the cholesterol biosynthesis pathway. BRCC36 stabilizes HMGCR by removing K63-linked polyubiquitin chains from the protein, thereby preventing its ubiquitin-proteasomal degradation. HMGCR plays an important role in regulating ferroptosis and pyroptosis in HCC cells, with its sublocalization to mitochondria and endoplasmic reticulum determining the mode of cell death. Through deubiquitinating HMGCR, BRCC36 suppresses ferroptosis and promotes pyroptosis in HCC cells [91,92,93].

**Figure 3:**
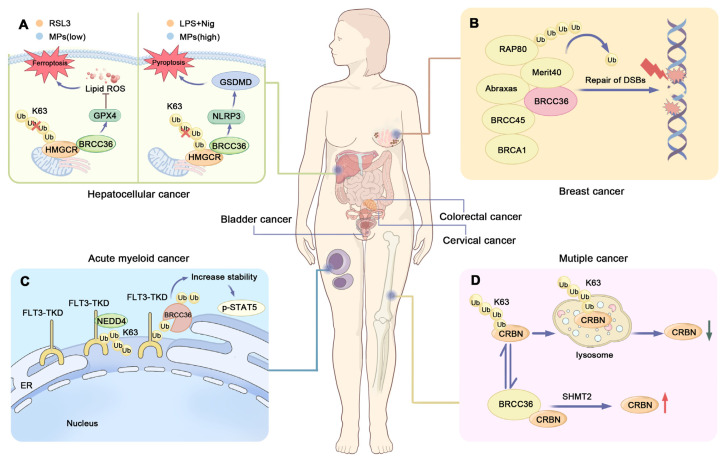
BRCC36 and carcinomas. (**A**) Model diagram of BRCC36 deubiquitinating HMGCR to regulate the interaction between ferroptosis and pyroptosis. BRCC36 stabilizes HMGCR protein by removing the K63 polyubiquitin chain. When cells are exposed to the ferroptosis inducer RSL3 or low concentrations of MPs, HMGCR is mainly localized in mitochondria and can prevent ferroptosis by regulating the activity of GPX4 to prevent the accumulation of lipid peroxides. When cells were exposed to LPS and Nig, or high concentrations of MPs, HMGCR was mainly localized in the endoplasmic reticulum, and this localization affected the assembly of GSDMD—N proteins in the membrane, which in turn led to cellular focal death. (**B**) Modeling of BRCA1-A complex recruitment to DNA damage sites. RAP80 and BRCA1 form the BRCA1-A complex, and RAP80 is responsible for recruiting BRCA1 to DNA double-strand breaks (DSBs). And defective or abnormal DSB repair mechanism is closely related to the development of breast cancer. (**C**) Schematic representation of the regulation of cellular function by FLT3-ITD and BRCC36. BRCC36 removes the K63-connected polyubiquitin chain on FLT3-ITD, thereby improving protein stability and downstream signaling. (**D**) Schematic representation of CRBN regulation by BRCC36. BRCC36 in the BRISC complex protects CRBN from lysosomal degradation by specifically cleaving the K63-linked polyubiquitin chain on CRBN. (HMGCR—3-hydroxy-3-methylglutaryl-CoA reductase, LPS—Lipopolysaccharide, MPs—microplastics, NLRP3—Nucleotide-binding oligomerization domain-like receptor family pyrin domain-containing 3, GPX4—Glutathione Peroxidase 4, DSBs—DNA double strand breaks, FLT3—FMS-like Tyrosine Kinase 3, TKD—Tyrosine kinase domain, CRBN—Cereblon, SHMT2—Serine hydroxymethyltransferase 2).

Notably, both BRCC36 and HMGCR are highly expressed in HCC tissues and are associated with unfavorable patient outcomes [91,94]. Thiolutin (THL), a disulfide-containing antibiotic produced by *Streptomycetes*, acts as a zinc chelator. It inhibits BRCC36 by sequestering the catalytic Zn^2+^ ion within the enzyme’s active site, thereby disrupting the BRCC36-HMGCR interaction and suppressing HCC cell growth. These findings suggest that targeting BRCC36 may represent a promising therapeutic strategy for HCC (Figure 3A) [91,95]. Furthermore, inhibition of the BRISC complex has been shown to suppress multiple inflammatory signaling pathways in the liver, indicating its potential beneficial role in modulating liver disease progression [96].

#### 4.1.2. Breast Cancer

Breast cancer is one of the most common malignant tumors worldwide and a leading cause of cancer-related mortality [97]. DNA double-strand breaks (DSBs) represent highly deleterious DNA lesions that require accurate recognition and repair to prevent genomic instability, programmed cell death, or malignant transformation [98]. Homologous recombination (HR)-mediated repair of DSBs is essential for the survival of breast cancer cells [99]. The most common cause of HR-mediated DNA repair defects is BRCA1/2 mutation, resulting in DSBs [100]. The role of ubiquitination in DSB repair is well-established, with multiple E3 ubiquitin ligases being recruited to DSB sites [101]. Among the regulators involved is BRCC36, a DUB specifically targeting K63-linked ubiquitin chains [10]. BRCC36 is abnormally expressed in most breast tumors and has been identified as a key contributor to breast tumorigenesis and progression [102,103]. Therefore, genes involved in DSB repair and protein ubiquitination play key roles in breast cancer pathogenesis, as they directly influence the efficiency of the DNA repair pathway, thereby modulating therapeutic resistance and genomic instability in breast cancer cells [104,105].

Research has established that Breast Cancer Type 1 Susceptibility Protein (BRCA1) functions as a tumor suppressor, which is involved in the differentiation of mammary epithelial cells [64,106]. The BRCA1-A complex plays an essential role in recruiting BRCA1 to DNA damage sites, which is important for DNA damage repair. Consequently, mutations in genes encoding subunits of the BRCA1-A complex may impair BRCA1 function and increase susceptibility to breast cancer [10,102,104]. Spatial transcriptomics has revealed that the pre-neoplastic mammary microenvironment is remodeled in BRCA1/2 mutation carriers, and that germline variants in BRCA1 and BRCA2 constitute major risk factors for specific breast cancer subtypes. Tumours arising in BRCA1-mutation carriers are overwhelmingly basal-like, whereas those in BRCA2-mutation carriers are predominantly luminal. Consequently, the aetiology, biology and optimal treatment strategies differ markedly between individuals harbouring germline BRCA1 versus BRCA2 mutations [107]. BRCC36, an integral component of the BRCA1-A complex, facilitates its localization to DNA damage sites through RAP80-mediated SUMOylation and recognition of K63-linked ubiquitin chains [108,109]. The N-terminal region of RAP80 contains a SUMO-interacting motif (SIM, residues 41–43) and two tandem ubiquitin-interacting motifs (UIMs, residues 80–99 and 105–124) with specificity for K63-linked chains, which confer high-affinity binding to hybrid ubiquitin-SUMO chains [109,110,111,112]. These domains, connected via flexible linkers, mediate the recruitment of the BRCA1-A complex to DNA lesions and facilitate its structural integrity (Figure 3B) [64,67,102]. It is noteworthy that BRCC36 not only serves as a critical structural component within the RAP80 complex in the DNA damage response but may also contribute to the functionality of the BRCA1-A complex through its DUB activity [113].

Breast cancer encompasses multiple subtypes, among which triple-negative breast cancer (TNBC) accounts for approximately 20% of all cases and is characterized by high aggressiveness [114]. Epithelial–mesenchymal transition (EMT) is widely recognized as a process closely associated with tumorigenesis, contributing to tumor invasion, metastasis, and recurrence. As a result, EMT has been proposed as a hallmark of cancer progression, and targeting EMT-related pathways represents a promising therapeutic strategy. The Zinc Finger E-box Binding Homeobox 1 (ZEB1) is a key transcriptional regulator of EMT and plays a central role in driving this process [115,116]. Accumulating studies have highlighted the importance of ZEB1 in TNBC [117,118]. ZEB1 expression is significantly upregulated in TNBC tissues. Knockdown of BRCC36 increases ZEB1 ubiquitination and promotes its degradation via the ubiquitin-proteasome system. This regulatory mechanism promotes TNBC cell proliferation, migration, invasion, EMT, and metastasis, offering a novel direction for therapeutic intervention in breast cancer [119].

#### 4.1.3. Acute Myeloid Leukemia

Mutations in FMS-like Tyrosine Kinase 3 (FLT3), such as internal tandem duplications (ITDs), are detected in up to 23% of acute myeloid leukemia (AML) patients and are frequently associated with poor prognosis [120]. FLT3 represents one of the most commonly mutated genes in AML. ITD mutations constitute the most prevalent type of FLT3 alteration, accounting for approximately 25% of all AML cases [121,122]. Point mutations within the tyrosine kinase domain (TKD) of FLT3 also occur, although the prognostic implications of FLT3-TKD mutations remain controversial [121,123]. Proximity labeling techniques, such as TurboID, have revealed that BRCC36 specifically interacts with FLT3-ITD and enhances its stability and downstream signaling. Knockdown of BRCC36 resulted in reduced phosphorylation of Signal Transducer and Activator of Transcription 5 (p-STAT5) and decreased proliferation of FLT3-ITD-positive cells (Figure 3C). Similarly, the BRCC36 inhibitor THL specifically suppressed the proliferation of ITD-bearing cells and induced apoptosis. THL effectively impaired the survival of FLT3-ITD-driven leukemic cell lines and demonstrated synergistic effects with Quizartinib, a standard therapy used in AML [124,125].

IFNs are cytokines that, upon binding to cell surface receptors, initiate a cascade of downstream biochemical events. Engagement of type I and type II IFN receptors activates the canonical JAK-STAT signaling pathway [126,127]. It is noteworthy that the presence of K63-linked ubiquitin chains on IFNAR1, and the role of this ubiquitin topology in IFNAR1 endocytosis and degradation, have been well established [13,128,129]. The t(8;21)(q22;q22) translocation is the most common chromosomal abnormality in the M2 subtype of AML, leading to the formation of the AML1-ETO fusion oncogene [130]. BRCC36 mutations exhibit a selective distribution in t(8;21)(q22;q22) AML. In clinical practice, AML patients harboring BRCC36 mutations (loss of ubiquitinase activity) are associated with a favorable prognosis. This may be attributed to the loss of BRCC36-mediated deubiquitinating activity toward IFNAR1, which consequently attenuates IFN signaling. Furthermore, inactivation of BRCC36 may impair DNA damage repair, rendering leukemic cells more sensitive to chemotherapy-induced apoptosis and thereby improving treatment efficacy [131,132].

#### 4.1.4. Multiple Myeloma

Multiple myeloma (MM) is the second most common hematologic malignancy and is commonly treated with immunomodulatory drugs (IMiDs) and proteasome inhibitors (PIs) [120]. IMiDs such as lenalidomide (Len) represent a cornerstone of current MM therapy. However, the development of Len resistance remains a major clinical challenge [133,134]. Cereblon (CRBN) acts as a substrate receptor of the cullin-RING E3 ubiquitin ligase complex, where it recruits specific protein substrates for ubiquitination [135,136]. In MM, Len binds to CRBN and promotes the ubiquitination and subsequent degradation of two lymphoid transcription factors, IKZF1 and IKZF3, which are essential for myeloma cell survival. This mechanism ultimately inhibits myeloma cell proliferation. However, this process can be suppressed by BRCC36 [137,138].

BRCC36 is an interacting protein of CRBN that specifically cleaves K63-linked polyubiquitin chains on CRBN (Figure 3D), thereby protecting it from autophagy-lysosome degradation [102]. Notably, the combination of Len with Serine hydroxymethyltransferase inhibitor 1 (SHIN1)—a small-molecule compound that binds to SHMT2, a subunit of the BRISC complex—further enhances the sensitivity of myeloma cells to Len [139]. As an inhibitor of SHMT2, SHIN1 disrupts the SHMT2-BRCC36 interaction, leading to increased CRBN protein stability and augmented anti-myeloma efficacy of Len [102].

#### 4.1.5. Bladder Cancer

Bladder cancer (BCA) is one of the most common malignancies of the urinary system [140]. While it presents a significant clinical burden, it is also considered a preventable disease due to the presence of modifiable risk factors [141]. Nuclear Factor kappa B (NF-κB) plays a central role in chronic inflammation and cancer development [142]. Bioinformatic analyses and experimental validation have revealed that BRCC36 is significantly upregulated in BCA. Knockdown of BRCC36 inhibits the proliferation and migration of BCA cells and leads to marked inactivation of the NF-κB signaling pathway. Furthermore, elevated BRCC36 expression promotes tumorigenesis in BCA by activating NF-κB signaling through targeting TRAF2. These findings identify BRCC36 as a potential therapeutic target for the treatment of BCA [143].

#### 4.1.6. Colorectal Cancer

Colorectal cancer (CRC) is a leading cause of cancer-related mortality worldwide [144]. Colorectal adenocarcinoma (COAD), the predominant histological subtype of CRC, is often characterized by inconspicuous early symptoms and unfavorable prognosis [145,146]. EMT, a process of cellular reprogramming, along with its reverse process, mesenchymal–epithelial transition (MET), has been implicated in CRC progression [147]. Studies have demonstrated that BRCC36 expression is elevated in COAD tissues compared to adjacent normal tissues. BRCC36 promotes migration, invasion, and EMT in COAD cells by stabilizing MET protein, suggesting its potential role as a diagnostic and therapeutic biomarker for COAD [145].

#### 4.1.7. Cervical Cancer

Cervical cancer (CCA) is the third most common malignancy in women worldwide, following breast and colorectal cancer, and is associated with high incidence and mortality rates [148]. Snail, a transcription factor, plays a critical role in driving EMT in CCA cells [115]. BRCC36 expression is significantly upregulated in CCA tissues compared to normal cervical epithelium. Its expression level correlates positively with clinical stage, pathological grade, and shorter overall survival in CCA patients. Elevated BRCC36 expression promotes EMT and enhances malignant behaviors of CCA cells by modulating EMT-related markers and members of the Snail family. Conversely, knockdown of BRCC36 suppresses the EMT process, indicating its functional importance in disease progression [149,150].

#### 4.1.8. Prostate Cancer

Genome instability, often arising from mutations in genes associated with DNA repair, serves as both the cause of carcinogenesis and a target for precision therapy [151,152,153]. Poly (ADP-ribose) polymerases (PARPs), which respond to DNA damage, have thus been exploited as therapeutic targets [154]. PARP inhibitors (PARPi) achieve this by inhibiting PARP, leading to its entrapment on DNA, subsequent replication arrest, and the generation of double-strand breaks. This synthetic lethal approach is particularly effective against BRCA-mutant cancers, such as cervical cancer and breast cancer [151,155]. While demonstrating benefit in prostate cancer patients who have exhausted conventional therapies, PARPi efficacy is ultimately limited by acquired resistance. Transcriptomic analysis of three olaparib-resistant cell lines revealed a significant upregulation of BRCC3, implicating its overexpression as a key mechanism underlying acquired resistance of PARPi in prostate cancer and a promising therapeutic target for its reversal [156,157].

### 4.2. Roles of BRCC36 in Non-Neoplastic Diseases

#### 4.2.1. Chronic Kidney Disease

Vascular calcification (VC) is a common complication of chronic kidney disease (CKD) and represents an active process involving multiple pathogenic factors and distinct mechanisms, notably chronic inflammation and oxidative stress [158,159,160,161]. Additionally, the WNT/β-catenin signaling pathway has been implicated in CKD-related vascular calcification and mineral bone disorders [150,162]. Studies have shown that BRCC36 expression is downregulated in CKD-induced VC and negatively correlates with the severity of calcification. BRCC36 reduces K63-linked ubiquitination of β-catenin, thereby inhibiting its nuclear translocation and transcriptional activity. Through suppression of the Wnt/β-catenin pathway, BRCC36 exerts anti-calcific effects [163,164].

**Figure 4:**
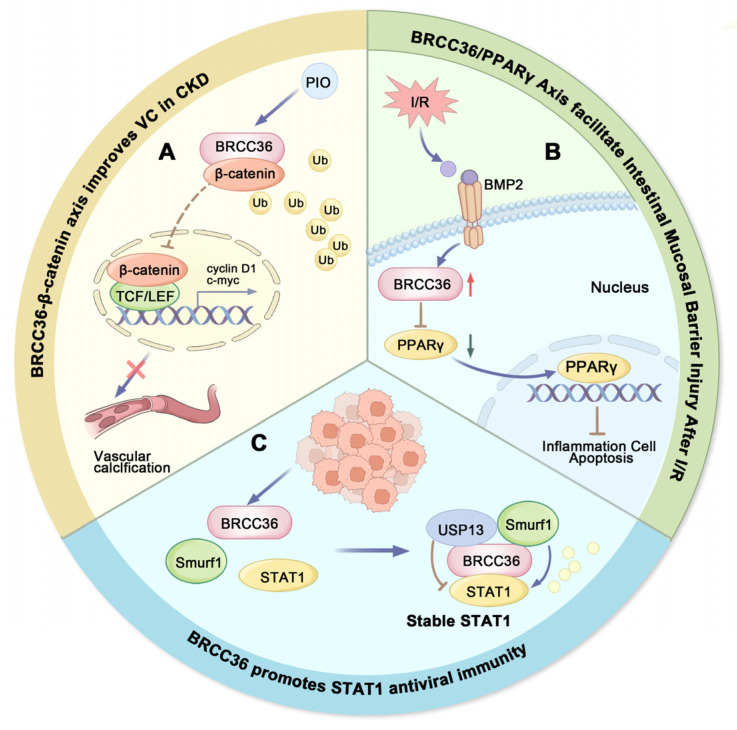
Roles of BRCC36 in non-tumorigenic diseases. (**A**) Pattern plot of BRCC36-β-catenin axis improving vascular calcification. BRCC36-mediated deubiquitination inhibits the Wnt/β-catenin signaling, and pioglitazone (PIO) can attenuate VC by upregulating BRCC36 expression. (**B**) Schematic representation of BRCC36/PPARγ axis promoting intestinal mucosal barrier damage after I/R. I/R stress induced upregulation of BRCC36 and inhibited the downstream PPARγ pathway, ultimately leading to gut barrier dysfunction. (**C**) Schematic representation of BRCC36 promoting STAT1 antiviral immunity. BRCC36 maintains cellular STAT1 protein stability against viruses by forming an equilibrium complex with Smurf1 and USP13. (PIO-Pioglitazone, I/R-Ischemia–reperfusion, BMB2-Deubiquitinases as novel therapeutic targets for diseases).

Pioglitazone (PIO), an agonist of Peroxisome Proliferator-activated Receptor gamma (PPARγ), confers renal protection by enhancing antioxidant capacity and mitigating inflammation [165,166]. Furthermore, PIO attenuates β-glycerophosphate (β-GP)-induced calcification in rat vascular smooth muscle cells (VSMCs) by inhibiting Wnt/β-catenin signaling [167]. It also partially alleviates VC by upregulating BRCC36 expression and downregulating β-catenin. These findings suggest that BRCC36 may serve as a key mediator of PIO’s anti-calcification actions. The role of BRCC36 in CKD-associated VC and its underlying molecular mechanisms provide a rationale for developing novel therapeutic strategies against VC. Both BRCC36 and β-catenin represent potential therapeutic targets for preventing the progression of VC in CKD patients (Figure 4A) [164].

#### 4.2.2. Ischemia/Reperfusion Injury

Cerebral ischemia–reperfusion (I/R) injury is a cerebrovascular disorder in which inflammation serves as a critical pathological component [168]. The NOD-like Receptor Protein 6 (NLRP6) has been shown to exert pro-inflammatory effects during cerebral I/R injury, suggesting its potential as a therapeutic target [169]. Previous studies have demonstrated that BRCC36 is upregulated following cerebral I/R injury and is primarily localized in neurons. It interacts with NLRP6, modulates its ubiquitination status, and promotes the assembly of the NLRP6 inflammasome. The role of BRCC36 in I/R injury and its mechanism of regulating neuroinflammation and focal necrosis via the NLRP6 inflammasome provides a mechanistic basis for developing novel therapeutic strategies against cerebral I/R injury. Therefore, BRCC36 may represent a promising therapeutic target for reducing brain damage and improving neurological recovery after I/R injury [3,14].

Intestinal I/R injury is a common pathophysiological process encountered during surgical procedures, which can lead to acute intestinal obstruction, transplantation-related complications, abdominal trauma, shock, and other conditions associated with acute vascular insufficiency, high morbidity, and mortality [170]. In intestinal I/R injury, activation of NF-κB is involved in the induction of Bone Morphogenetic Protein (BMP) signaling, which suppresses the expression of tight junction proteins and disrupts the intestinal mucosal barrier [171]. BRCC36 plays a critical role in this process by inhibiting the PPARγ signaling pathway (Figure 4B), thereby exacerbating BMP2-induced damage to the mucosal barrier. Furthermore, upregulation of BRCC36 downregulates tight junction protein expression, promotes apoptosis of intestinal epithelial cells, and enhances the production of inflammatory cytokines, collectively accelerating intestinal barrier dysfunction. Thus, targeting BRCC36 may represent a promising therapeutic strategy for mitigating intestinal I/R injury (Figure 4B) [172].

#### 4.2.3. Dry Eye Disease

Dry eye disease (DED) is a common autoimmune disorder characterized by ocular surface inflammation, reduced tear secretion, and increased tear osmolarity [173]. Members of the NOD-like Receptor (NLR) family contribute to innate immune responses by participating in diverse cellular processes [174]. The NOD-like Receptor Protein 3 (NLRP3) inflammasome represents a key component of the innate immune system and has been implicated in various pathological conditions, including infectious and autoimmune diseases [17,175]. BRCC36 serves as a critical regulator of ocular surface inflammation in DED. It exacerbates inflammatory responses by activating the NLRP3 inflammasome while simultaneously activating caspase-8, which then suppresses NLRP6 through an as-yet-unknown mechanism. Oxidative stress and mitochondrial DNA damage are important triggers of BRCC36 activation. Under the pathological conditions of DED, hyperosmolarity and desiccating stress lead to increased production of reactive oxygen species (ROS), which in turn activates BRCC36. Targeting the BRCC36/NLRP3/NLRP6 axis may therefore offer a promising therapeutic strategy to alleviate ocular inflammation and improve surface health in DED [176,177].

#### 4.2.4. Pulmonary Arterial Hypertension

Pulmonary arterial hypertension (PAH) is a disorder characterized by elevated pulmonary arterial pressure and pathological remodeling of pulmonary vessels, often accompanied by varying degrees of perivascular inflammatory infiltration [178]. In the presence of mutations in the Bone Morphogenetic Protein Receptor type 2 (BMPR2), inflammatory pathology is exacerbated. Transforming growth factor-beta (TGF-β), a key regulator of immune tolerance and an anti-inflammatory factor, plays an important role in this process. The pathogenesis of PAH involves an imbalance between BMP and TGF-β signaling pathways [102,179,180].

BRCC36 enhances BMP signaling by deubiquitinating ALK2, a critical component of the BMP pathway, thereby inhibiting the proliferation and migration of pulmonary arterial smooth muscle cells (PASMCs) and promoting pulmonary vascular homeostasis. Downregulation of BRCC36 leads to suppression of BMP signaling and hyperactivation of the TGF-β pathway, which in turn drives pathological vascular remodeling and PA progression. Therefore, targeted upregulation or activation of BRCC36 may represent a novel therapeutic strategy for PAH [181].

#### 4.2.5. Atherosclerotic Cardiovascular Disease

Atherosclerosis is a chronic inflammatory disease [182]. Interleukin-1β (IL-1β) and other members of the interleukin-1 cytokine family act as systemic inflammatory mediators involved in atherogenesis [183]. Inflammasomes play an essential role in the production of IL-1β, particularly the NLRP3 inflammasome, which has been strongly implicated in atherosclerosis [184,185].

Clonal hematopoiesis represents a major independent risk factor for atherosclerotic cardiovascular disease, thrombosis, and heart failure, and is associated with enhanced macrophage inflammasome activation [186]. The TET family proteins, TET1 and TET2, as well as TET3, function as dioxygenases that regulate DNA demethylation. Among them, TET2 is widely recognized as a critical regulator of normal hematopoiesis, especially myelopoiesis. Mutations and functional impairments in TET2 contribute to the development of various hematological malignancies [187].

BRCC36 activity is closely linked to cholesterol metabolism, and its inhibition has been shown to attenuate atherosclerotic progression. BRCC36-mediated NLRP3 inflammasome activation is significantly enhanced within atherosclerotic plaques, particularly in TET2-deficient macrophages. Pharmacological inhibition of BRCC36 reduces inflammasome activation, diminishes atherosclerotic lesion size, and improves plaque stability. Thus, BRCC36 represents not only a key pathophysiological mediator but also a promising therapeutic target in cardiovascular disease [188,189].

#### 4.2.6. Asthma

Asthma is a heterogeneous inflammatory disease characterized by persistent airway inflammation, bronchial hyperresponsiveness, and airflow limitation [190]. BRCC36 expression is significantly upregulated in the bronchial epithelium of asthma patients as well as in murine models of asthma. By removing K63-linked ubiquitin chains from NLRP3, BRCC36 enhances the activation of the NLRP3 inflammasome, thereby promoting airway inflammation and pyroptosis. These findings indicate that BRCC36 serves not only as a key regulator in the pathogenesis of asthma but also as a potential therapeutic target for novel anti-asthma strategies [176,191,192].

#### 4.2.7. Pulpitis

The dental pulp is prone to a painful inflammatory condition known as pulpitis. In this context, BRCC36 primarily promotes inflammatory responses and exacerbates pulp tissue damage through activation of the NF-κB signaling pathway. Inhibition of BRCC36 activity significantly attenuates the severity of pulpitis, reduces the release of inflammatory cytokines, and mitigates inflammatory cell infiltration [193].

#### 4.2.8. Moyamoya Disease

Moyamoya disease is a rare cerebrovascular disorder characterized by progressive bilateral stenosis of the internal carotid arteries, accompanied by the development of fragile compensatory collateral vessels, known as “moyamoya” vessels, which often lead to ischemic or hemorrhagic stroke [194]. BRCC36 plays a critical role in angiogenesis, and its deficiency has been associated with impaired vascular development, suggesting a potential involvement in the pathological mechanisms underlying moyamoya vasculopathy [102].

#### 4.2.9. Viral Infections

Viral infections pose a serious threat to human health. Protein ubiquitination has been recognized as a critical regulatory mechanism in antiviral immunity, with both E3 ubiquitin ligases and DUBs playing extensive roles in host antiviral responses [195,196]. As a key mediator of IFN signaling, STAT1 (a transcription factor that promotes IFN-γ-induced immune responses) has been well established to perform essential functions in antiviral immune reactions [197]. BRCC36 modulates the ubiquitination status and stability of STAT1 by forming a regulatory complex with USP13 and Smurf1 (Figure 4C), which fine-tunes its activity in immune signaling [198].

HIV-1 is a lentivirus that encodes three structural proteins common to all retroviruses (Gag, Pol, and Env), as well as six accessory proteins unique to HIV-1 (Tat, Rev, Vpr, Vif, Vpu, and Nef) [199]. Among these, HIV-1 Tat and Rev play critical early regulatory roles in the viral life cycle. Tat, in particular, has been a major focus of HIV research, and its transcriptional activity is known to be stimulated by ubiquitination [200]. The protein level of HIV-1 Tat is modulated through K63-linked ubiquitination mediated by the SHMT2-BRCC36 deubiquitinating enzyme complex. This modification prolongs Tat’s half-life but concurrently inactivates its transcriptional function [9,201].

## 5. Knowledge Gaps and Future Perspectives

Research on BRCC36 and its associated complexes in disease has advanced yet remains marked by significant knowledge gaps. While it is established that the deubiquitinase activity of BRCC36 depends on SHMT2, the precise mechanism of this cooperation is unclear. For instance, whether SHMT2 recruits BRCC36 to substrates through competitive binding or post-translational modifications is unknown. Furthermore, BRCC36 functions within two distinct complexes, BRISC and BRCA1-A, which are typically studied in isolation, with BRISC in modulating drug sensitivity in multiple myeloma and BRCA1-A in DNA damage repair in breast cancer. However, the potential crosstalk between these complexes within a single tumor type, and how their coordinated expression or activity influences oncogenesis, is unexplored. The pro-inflammatory role of BRCC36 adds another layer of complexity. It can enhance inflammation via either IFN signaling or NLRP3 inflammasome activation, but the determinants of this pathway selectivity in different diseases are undefined.

Drug development faces considerable hurdles due to the conserved structure of the JAMM/MPN metalloprotease domain, which complicates the creation of selective inhibitors. There are few existing compounds and they require thorough evaluation of their in vivo stability, toxicity, and pharmacokinetics. A critical future goal is to design inhibitors that can distinguish between the BRISC and BRCA1-A complexes for precise intervention. Therefore, interdisciplinary efforts are essential to translate basic research on BRCC36 into applications in precision medicine.

## 6. Conclusions

Deubiquitinating enzymes (DUBs) have attracted considerable research interest due to their crucial roles in maintaining cellular homeostasis by removing ubiquitin or cleaving ubiquitin chains from substrate proteins. DUBs regulate key signaling pathways such as NF-κB, PI3K/Akt/mTOR, and MAPK, and are implicated in a wide range of diseases, including cancer, neurodegenerative disorders, cardiovascular conditions, inflammation, and developmental abnormalities. Among DUBs, BRCC36 has recently emerged as a functionally significant component. This review has summarized the subcellular localization, functional characteristics, site-specific mutations, and complex assembly of BRCC36, along with a detailed discussion of its roles across various diseases. Encoded by the BRCC3 gene, the K63-linkage-specific deubiquitinase BRCC36 primarily incorporates into two distinct complexes, BRCA1-A and BRISC, which differ in subunit composition and biological functions, and are associated with multiple pathological processes. In oncology, dysregulated expression of BRCC36-containing complexes is closely linked to tumor initiation, progression, invasion, and metastasis in cancers such as breast cancer, hepatocellular carcinoma, and leukemia. BRCC36 expression levels may also serve as a prognostic biomarker in certain malignancies. Beyond cancer, BRCC36 contributes significantly to non-oncological diseases; for example, it modulates inflammatory responses via regulation of NF-κB signaling and supports antiviral immunity by maintaining STAT1 stability.

Given these multifaceted functions, targeting the BRCC36 complex represents a promising therapeutic strategy. BRCC36, a DUB with a well-defined active site, represents a druggable target. The existence of a known inhibitor, THL, serves as a reference for developing next-generation agents with superior potency and reduced side effects. Furthermore, observed synergistic effects in combination therapies underscore the potential of leveraging BRCC36 inhibitors within broader drug regimens. Beyond its catalytic function, BRCC36 can promote tumorigenesis through non-canonical, activity-independent mechanisms. For instance, in bladder cancer, BRCC36 enhances NF-κB pathway activation and drives inflammation and oncogenesis by directly interacting with TRAF2. This finding reveals a novel therapeutic avenue that blocks the interaction of BRCC36 and TRAF2 and combines with NF-κB pathway inhibitors. The development of inhibitors, activators, or modulators specifically directed against BRCC36 or its complexes may offer novel treatment avenues for related diseases. Looking forward, research on the BRCC36 complex remains highly promising. Although progress has been made in understanding its roles in DNA damage repair, signal transduction, and cell cycle regulation, many mechanistic details require further elucidation. Current drug development efforts targeting BRCC36 are still in early stages, underscoring the need for deeper structural and functional insights to facilitate the design of highly specific and effective therapeutic agents.

## Figures and Tables

**Table 1:** Subunits of the BRISC and BRCA1-A complexes.

Subunit	Common Alias	Assembly	Characteristic	Related Diseases	Refs
BRCC36	BRCC3	BRISC BRCA1-A	Enzymatic activity	Cancer Inflammation	[14]
Abraxas	ABRA1	BRCA1-A	Carries a nuclear import signa	Breast cancer	[15,16]
ABRO1	ABRAXAS 2 KIAAO157	BRISC	Contain a JAMM/MPN −domain	Cancer Myocardial Infarction	[17]
BRCC45	BRE	BRISC BRCA1-A	Binds ubiquitin	Cancer Brain Glioma	[18]
MERIT40	NBA1	BRISC BRCA1-A	Binds ubiquitin	Cherubism Cutis Laxa	[11,19]
RAP80	UIMC1	BRCA1-A	An extended, largely unstructured protein	Cancer Fanconi Anemia	[20,21]
SHMT2	SHMT	BRISC	A receptor-associated protein	Cancer Hepatitis	[22]

**Table 2:** The roles of DUBs in a variety of human diseases.

Family	DUBs	Function	Related Diseases	Refs
USP	USP1	Regulation of DNA damage repair	Fanconi Anemia Cancer	[40,41]
	USP7	Protein stabilizer and regulate processes	Cancer Viral Infection	[42,43]
	USP14	Central regulator of the 26S proteasome	Cancer Viral Infection	[44,45]
	USP18	Negative regulation of type I interferon signaling pathway	Cancer Viral Infection	[46,47]
JAMM	BRCC36	DNA damage repair and regulation of inflammatory responses	Cancer Viral Infection	[48]
	AMSH	Receptor endocytosis and signaling pathway regulation	Neurodegenerative Disease Cancer	[49]
	Rpn11	A variety of proteasome-dependent biological processes	NAFLD Cancer	[7]
UCH	UCHL1	Oxidative stress homeostasis, protect neuronal function	Neurodegenerative Diseases Cancer	[50]
	BAP1	Biological roles in metabolism, cancer	Cancer Kury-Isidor Syndrome	[51]
OTU	OTUB1	DNA repair, immunosuppression, tumorigenesis	Cancer Autoimmune diseases	[52]
	OTUD5	Immune response, DNA damage repair	Cancer Inflammatory diseases	[53]
	A20	Anti-inflammatory, regulation of cell death	Autoimmune Disease Cancer	[54]
MJD	ATXN3	Regulate transcription Control genome stability after DNA damage	Cerebellar Ataxia Cancer	[55]
MINDY	MINDY1	Regulation of proteostasis through K48-specific deubiquitinating enzyme activity	Cancer	[56]
ZUP1	ZUP1	Promote genomic stability	Cancer	[57]

## Data Availability

Not applicable.

## Abbreviations

BRCC36	BRCA1-BRCA12 containing complex subunit 3.
DUB	Deubiquitinating enzyme.
JAMM	JAB1/MPN/Mov34 metalloenzymes (JAMM) family.
SHMT2	Serine hydroxymethyltransferase 2.
Ub	Ubiquitin.
E1	Ubiquitin activating enzyme.
E2	Ubiquitin conjugating enzyme.
E3	Ubiquitin ligase.
IFN	Interferon.
NLRP3	Nucleotide-binding oligomerization domain-like receptor family pyrin domain-containing 3.
LPS	Lipopolysaccharide.
HCC	Hepatocellular carcinoma.
THL	Thiolutin.
DSBs	DNA double-strand breaks.
HR	Homologous recombination.
BRCA1	Breast Cancer 1.
UIM	Ubiquitin-Interacting Motif.
TNBC	Triple-negative breast cancer.
EMT	Epithelial–mesenchymal transition.
ZEB1	Zinc Finger E-box Binding Homeobox 1.
FLT3	FMS-like Tyrosine Kinase 3.
ITDs	Internal tandem duplications.
AML	Acute myeloid leukemia.
TKD	Tyrosine kinase domain.
IMiDs	Immunomodulatory drugs.
PLP	Pyridoxal 5′ -phosphate.
NF-Κb	Nuclear factor kappa-B.
VWA	Von Willebrand factor (vWF) type A domain.
BMP2	Bone morphogenetic protein 2.
CKD	Chronic kidney disease.
PIO	Pioglitazone.
HMGCR	3-Hydroxy-3-methylglutaryl-coenzyme A reductase.
VC	Vascular calcification.
I/R	Ischemia–reperfusion.
PPARγ	Peroxisome proliferator-activated receptor γ.
MM	Multiple myeloma.
CRBN	Cereblon.
SHIN1	Serine hydroxymethyltransferase inhibitor 1.
BCA	Bladder cancer.
CRC	Colorectal cancer.
COAD	Colorectal adenocarcinoma.
MET	Mesenchymal–epithelial transition.
CCA	Cervical cancer.
PARPs	Poly (ADP-ribose) polymerases.
PARPi	PARP inhibitors.
GPX4	Glutathione Peroxidase 4.
MPs	Microplastics.
β-GP	β-glycerophosphate.
VSMCs	Vascular smooth muscle cells.
NLRP6	NOD-like Receptor Protein 6.
DED	Dry eye disease.
ROS	Reactive Oxygen Species.
PAH	Pulmonary arterial hypertension.
TGF-β	Transforming growth factor-beta.
IL-1β	Interleukin-1β.
